# Taabo Multigenerational Birth Cohort in Côte d'Ivoire: Protocol for Establishing a Longitudinal Multigenerational Birth Cohort to Guide Health Policy

**DOI:** 10.2196/70771

**Published:** 2025-10-07

**Authors:** Siaka Koné, Nicole Probst-Hensch, Daouda Dao, Jürg Utzinger, Martial Laubet, Abou Marie-Chantal Tobo, Günther Fink

**Affiliations:** 1 Swiss Tropical and Public Health Institute, Allschwil, Switzerland; 2 University of Basel, Basel, Switzerland; 3 Centre Suisse de Recherches Scientifiques, Adiopodoumé, km 17, Route de Dabou, Abidjan, 01 BP 1303, Côte d'Ivoire, 225 0749069627; 4 Université Félix Houphouët-Boigny, Abidjan, Côte d'Ivoire

**Keywords:** birth, cohort study, Côte d'Ivoire, multigenerational cohort, Taabo HDSS, Taabo Health and Demographic Surveillance System

## Abstract

**Background:**

A large number of sociodemographic, economic, environmental, and psychosocial changes have contributed to the epidemiological transition of African countries and fundamentally shifted the primary drivers of health. Cohort studies are essential for understanding and improving population health but remain scarce in sub-Saharan Africa.

**Objective:**

The main objective of the Taabo Multigenerational Cohort (MGC) project is to establish a large, regionally representative multigenerational cohort. The cohort will be established within the Taabo Health and Demographic Surveillance System (HDSS) and used for studying the life course and intergenerational dynamics of disease in the south-central part of Côte d’Ivoire.

**Methods:**

The Taabo MGC project focuses on children born between January 1, 2024, and December 31, 2025, in the Taabo HDSS, as well as their parents, grandparents, and great-grandparents. Eligible women and their children are enrolled during pregnancy, and women who do not report during pregnancy are enrolled after the birth. After enrollment of pregnant women, biological ancestors of the index child who are still alive and living in the study area are recruited into the cohort. The cohort is expected to enroll at least 3000 pregnant women and their children, as well as the infants’ fathers, grandparents, and great-grandparents, with an expected sample size of approximately 15,000 individuals. To ensure the entire local population is covered in this study, we will also include 100 adults without children. The baseline assessments cover data on demographics, household wealth, tobacco and alcohol consumption, diet, physical activity, health history, quality of life, environmental exposures, depression, anxiety, stress, resilience, obstetric history, birth outcomes, cognitive function, and, for older adults, physical performance. We will also collect anthropometric measurements, blood pressure, and hemoglobin levels and check for *Plasmodium* infection (the causative agent of malaria) among all participants.

**Results:**

As of December 2024, the Taabo MGC project has enrolled 3239 women and 6501 family members. The enrollment of pregnant women and their children’s biological ancestors will continue until the end of 2025, aiming to reach at least 15,000 adults.

**Conclusions:**

The Taabo MGC project is designed to become one of the largest cohort studies in the region. Once established, the Taabo MGC project should become a platform for future observational and interventional studies at the local level and contribute to the much-needed evidence base on lifetime disease risk in this part of Côte d’Ivoire. We hope that our work will stimulate research and guide health policy elsewhere in sub-Saharan Africa.

## Introduction

### Background

Côte d’Ivoire, like most countries in sub-Saharan Africa (SSA), faces several major health challenges. Despite being one of the fastest-growing economies on the subcontinent [1], life expectancy in Côte d’Ivoire remains one of the lowest globally, with an average life expectancy of 59 years at birth in 2022 [2]. Low literacy, lack of quality health care for both newborns and mothers, and lack of accessible water and sanitation have been identified as the main drivers of low life expectancy [3,4]. While the burden of communicable diseases (CDs) continues to be high, rapidly rising noncommunicable diseases (NCDs) further challenge the health system [5]. The CDs causing the highest disease burden are malaria, tuberculosis, and HIV/AIDS. The NCDs causing the highest burden of disease are cardiovascular disease, chronic respiratory disease, cancer, and diabetes [6].

The Taabo Health and Demographic Surveillance System (HDSS) has been monitoring vital statistics in the south-central part of Côte d’Ivoire since 2009 [7]. Most recent estimates from the Taabo HDSS suggest that CDs caused 59% of all deaths reported, while NCDs were the primary cause in 19% of reported deaths. These NCD deaths were mostly due to cardiovascular diseases [8]. Capacity for NCD screening and management remains limited despite the high prevalence of prediabetes (47%), diabetes (8%), prehypertension (34%), and hypertension (31%) among adults aged 35 years and older [9-11]. In order to meet the ambitious health-related targets of the Sustainable Development Goals by 2030, high-impact and tailored interventions will be needed at the individual, household, community, and health system levels.

A growing body of evidence suggests that a substantial part of adult morbidity may be attributable to adversity in early life. The fetal origin hypothesis by Barker [12,13] postulates that inflammatory prenatal environments may induce molecular changes that predispose the fetus for (early) development of diseases in later life. To study such long-term relationships in low-income settings, detailed data on internal (genetic and biological) and external (eg, physicochemical, psychosocial dietary environment) exposomes are needed. Birth cohorts are ideal for such studies, as they allow an in-depth study of inter- and transgenerational traits that predetermine health outcomes over the life course [14-19]. While SSA has the highest burden of maternal, perinatal, and child deaths globally, birth cohorts that would allow life-course epidemiology and the use of biomarker research technologies remain scarce [20-22], limiting urgently needed research of the time- and context-specific risk factors underlying the high morbidity and mortality levels currently observed [23].

While the HDSS has been collecting data on all vital events (pregnancies, births, deaths, and migrations) as well as data on causes of death since 2009, data on biological, behavioral, environmental, genetic, and other risk factors have for the first time been collected systematically in the area in the context of the novel and innovative Côte d’Ivoire Dual Disease Burden (CoDuBu) cohort. The CoDuBu cohort has established the first population-based biobank in Côte d’Ivoire embedded into a broad exposome concept and study protocol [9-11,24].

The Taabo Multigenerational Cohort (Taabo MGC) project is completely independent of CoDuBu and focuses on differences in health behaviors and outcomes across generations. CoDuBu studies a population of approximately 1000 adults from both a behavioral and biomedical perspective, with rich biomarker and genetic data. The Taabo MGC project does not contain much biomedical information but instead collects basic behavioral and health outcomes for a much larger and diverse population of children and adults of all ages. The data and evidence emerging from this cohort will help identify high-impact and cost-effective interventions across specific time points in the life course and guide health policies at the country and regional level. The data will also be useful to further strengthen local research infrastructure and offer new opportunities for global comparative epidemiology.

### Objectives

The Taabo MGC aims to study the life course and intergenerational dynamics of disease in the south-central part of Côte d’Ivoire. Specifically, the Taabo MGC project aims to generate health data and evidence needed to guide health policy at the country and regional level. This will be accomplished by establishing a large, representative multigenerational cohort within the Taabo HDSS; investigating the prevalence of common health risks and diseases—including patterns of over- and undernutrition, anemia, *Plasmodium* infections, high blood pressure, adiposity, subjective well-being among adults, and mental health conditions; mapping and analyzing health care access across different age groups, sexes, and socioeconomic strata; and analyzing the intergenerational transmission of health risk factors and outcomes.

The goal of this protocol paper is to describe the scope and content of Taabo MGC as a reference for future studies using data from this cohort. We also hope that this study will generate additional interest in this cohort.

## Methods

### Study Setting

With a surface area of 322,462 km^2^, Côte d’Ivoire is a West African country bordered in the North by Mali and Burkina Faso, in the West by Liberia and Guinea, in the East by Ghana, and facing the Atlantic Ocean in the South. With a current estimated population of 29 million, Côte d’Ivoire’s population has more than doubled since 1990 [25]. In 2021, 52% of the population was urban, and 21.5% of the country’s total population lived in Abidjan as the largest urban area of the country [26]. Côte d’Ivoire is thought of as a subregional economic powerhouse, making a major contribution (40%) to the West African Economic and Monetary zone’s GDP and exports. Côte d’Ivoire’s economy is based on the primary sector, mainly agriculture (20% of GDP in 2021); the secondary sector (29% of GDP), mainly mining, energy, agri-food, and building and public works; and the tertiary sector (51% of GDP), mainly telecommunications, transport, trade, and financial activities [27].

**Figure 1.**
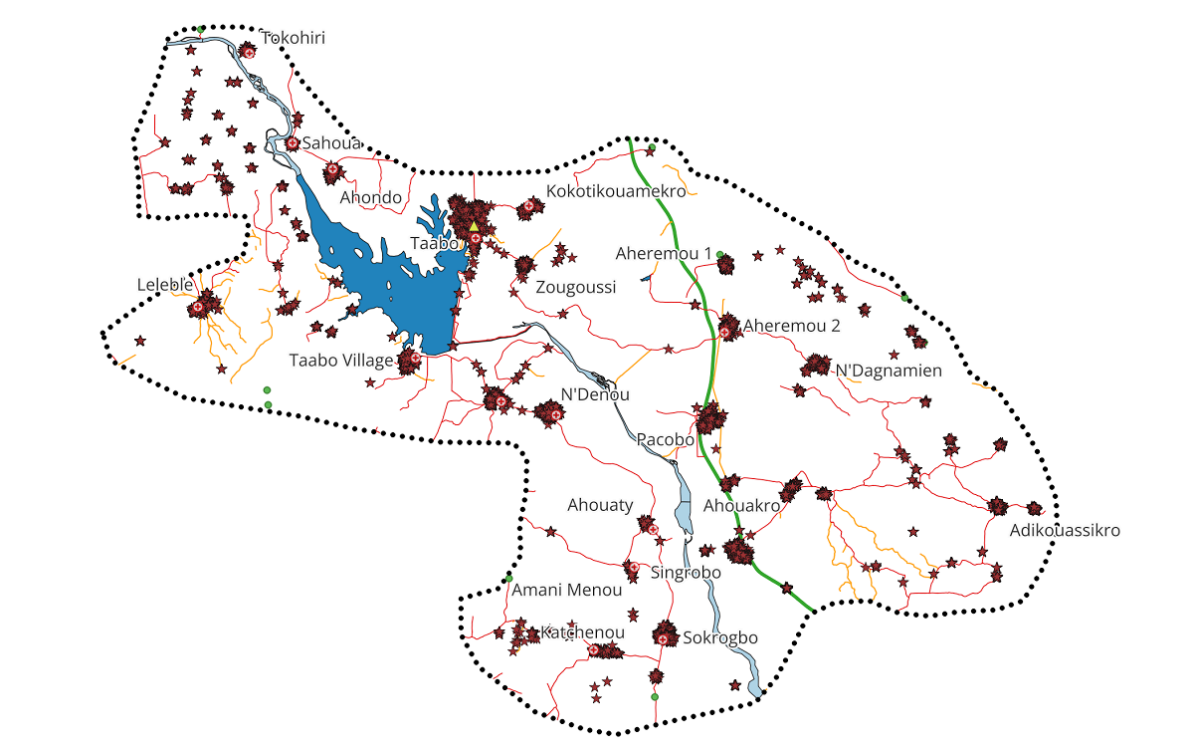
Health and demographic surveillance area in the south-central part of Côte d'Ivoire

Taabo MGC will be established in the Taabo HDSS, located around 160 km northwest of Abidjan. In 2018, the site had a total population of 46,847 spread across 14 villages (the small town of Taabo-Cité, 13 main villages, and more than 100 small hamlets). Since 2022, the surveillance zone has been extended to 21 localities illustrated in Figure 1 with a population of over 60,000. The study area is mainly rural, but the epidemiological transition is progressing rapidly. More detailed information on the Taabo HDSS can be found elsewhere [7].

### Study Design

The Taabo MGC is a prospective longitudinal health-focused cohort centered around pregnant mothers. The study was launched with the ambition to follow individuals over longer time periods and to study participant survival as well as a wide range of health outcomes. We will enroll all pregnant women over a 2-year enrollment period (November 2023 to September 2025) and then recruit their future children as well as all parents, grandparents, and great-grandparents who are still alive and living in the study area into the study.

### Inclusion Criteria

Pregnant women with expected delivery dates in 2024 or 2025 living in the Taabo HDSS area who provide written informed consent for enrollment and for contacting ancestral family members will be eligible for this study. Ancestral family members to be included in the study must also live in the Taabo HDSS area, provide informed consent to participate, and be biological ancestors of the index child. All pregnant women or ancestral family members who fail to meet any of these criteria will be excluded from the study.

### Study Procedures

**Figure 2.**
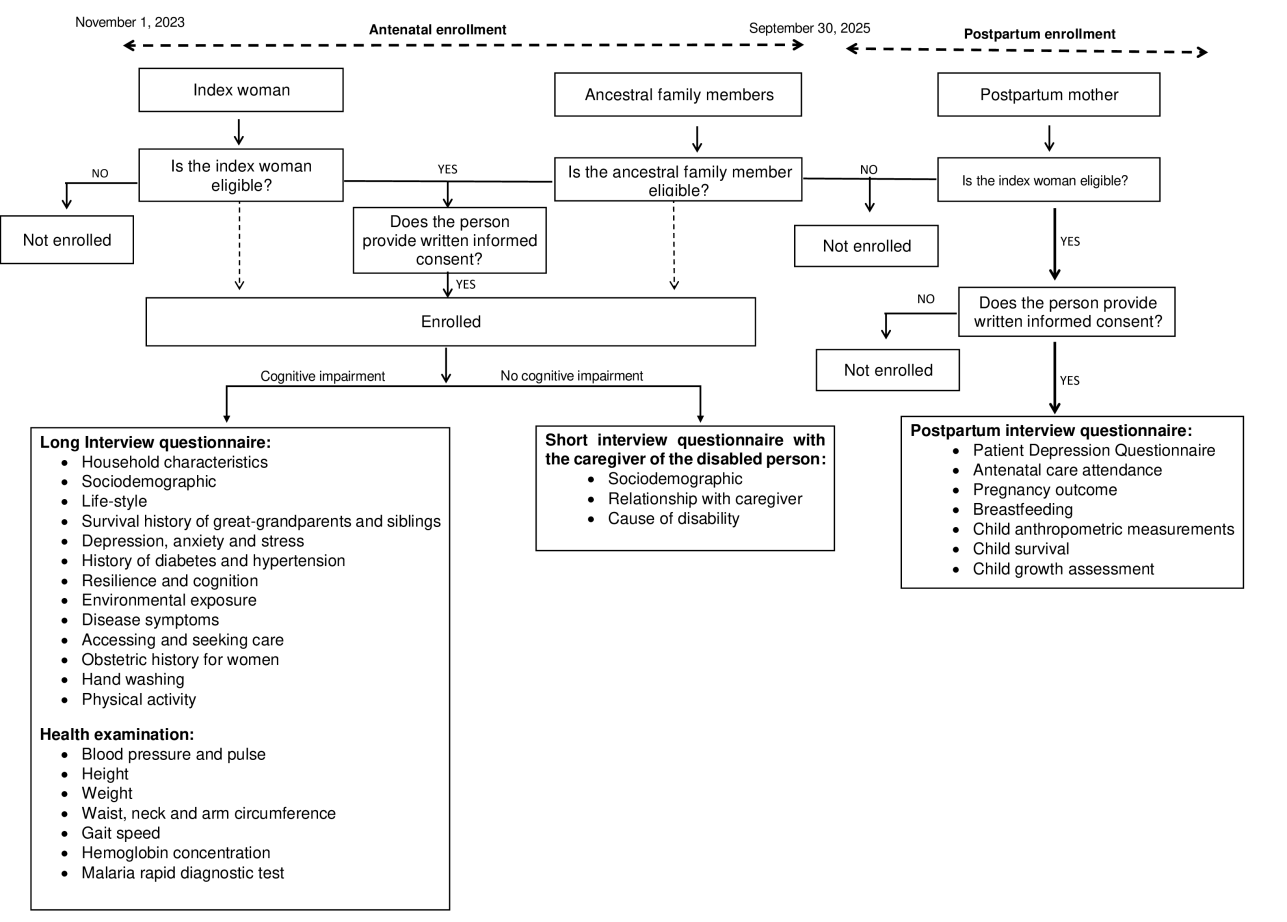
Study conceptual framework of the Taabo Multigenerational Cohort project in the south-central part of Côte d'Ivoire.

Figure 2 illustrates the main study procedures. All people who are not cognitively impaired who agree to take part in the study will undergo a lengthy interview questionnaire and a health examination. On the other hand, the caregivers of people experiencing cognitive impairment will be asked to complete a short questionnaire. With the phone number of the pregnant woman, each month, they are contacted to monitor the progress or term of the pregnancy. A postpartum questionnaire will be completed by each pregnant woman at the end of her pregnancy.

Ancestral biological family members who are related to more than one child will be enrolled only once. Similarly, the characteristics of a household with more than one participant will be collected only once.

### Participants

**Figure 3.**
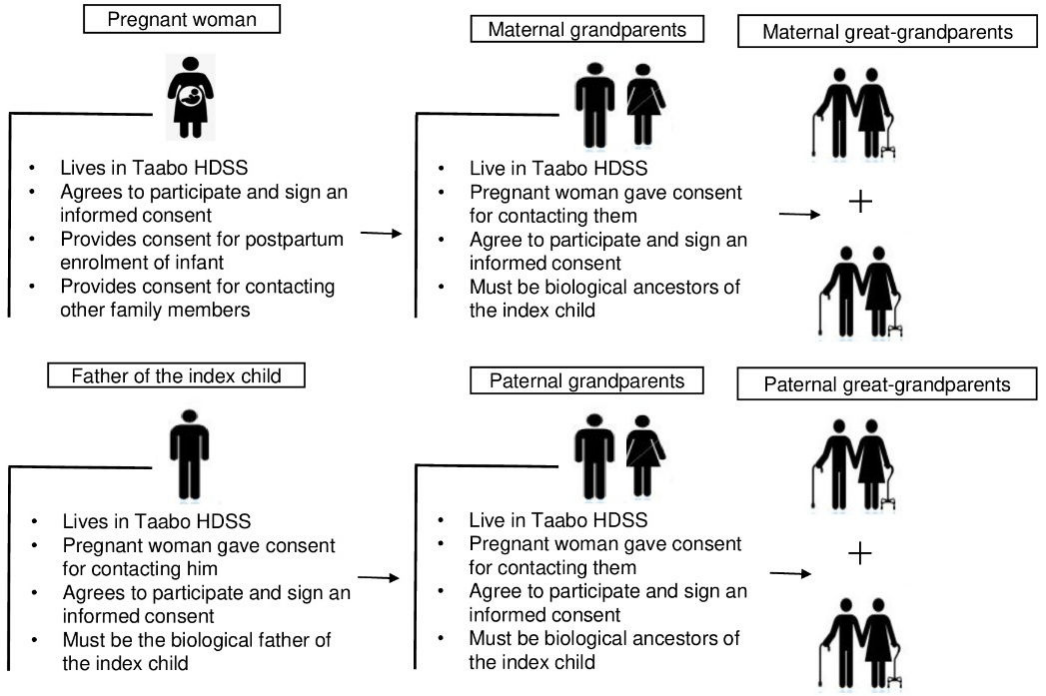
Enrollment pathway of the Taabo Multigenerational Cohort. HDSS: Health and Demographic Surveillance System.

The eligible members of each family will be recruited through its family tree, which will be built from the link between each member and the index child (Figure 3). The estimated sample size for the Taabo MGC is 3000 index families with an estimated 15,000 family members residing in the Taabo HDSS area. We anticipate enrolling 3000 pregnant women over a period of 2 years with an expected due date between January 1, 2024, and December 31, 2025. For each child born in the study period, we will also enroll the biological father as well as biological grandparents and great-grandparents if they are still alive and reside within the Taabo HDSS area. We plan on enrolling approximately 6000 parents, 6000 grandparents, and 3000 great-grandparents. In order to also cover childless adults living in this area, we will include an additional sample of approximately 50 adults aged between 40 years and 59 years and 50 adults aged 60 years or more in the study.

### Ethical Considerations

#### Overview

Ethics approval was obtained from the Ethics Committee Northwest and Central Switzerland (2023‐00049) and the National Ethics Committee for Life Sciences and Health in Côte d’Ivoire (139‐23/MSHPCMU/CNESVS-km). This research project will be carried out in compliance with the protocol, the Declaration of Helsinki [28], the Swiss Human Research Act, and the Swiss Human Research Ordinance [29], as well as other locally relevant regulations.

#### Compensation

No remuneration of participants is planned in this study. All subjects receive a summary report capturing the results of their physical examination, including blood pressure, height, and weight, as well as hemoglobin levels and *Plasmodium* infection for subjects consenting to these tests. Results will be explained in person and written on the summary report (Multimedia Appendix 1). If the test results suggest hypertension, anemia, or malaria infection, participants will be advised to go to their nearest health center for management.

#### Consent to Participate

Through an initial visit, the study team will explain the purpose and procedures of the study to each eligible participant. Written informed consent to participate will be obtained from all participants. Newborn consent will be sought from the pregnant woman. For participants aged less than 18 years, written informed consent to participate in the study will be sought from a parent or guardian in the household. Illiterate participants will be assisted by an additional witness. Participation is voluntary, and participants may withdraw from the study at any time or may refuse to answer any questions without having repercussions on their participation. If the participant decides to withdraw from the study, they may request that their information already obtained be destroyed and electronic data be deleted and not be used for evaluation. Confidentiality of information will be assured to the participants.

### Recruitment

As part of routine population surveillance, the field enumerators of the Taabo HDSS visit all the households within the Taabo HDSS 2 to 3 times per year to record vital events such as pregnancies, births and deaths, and migration. All pregnant women identified during routine surveillance will be invited by field enumerators to join the study. In addition, key informants will be recruited by the study team to allow for an early reporting of pregnancies.

**Figure 4.**
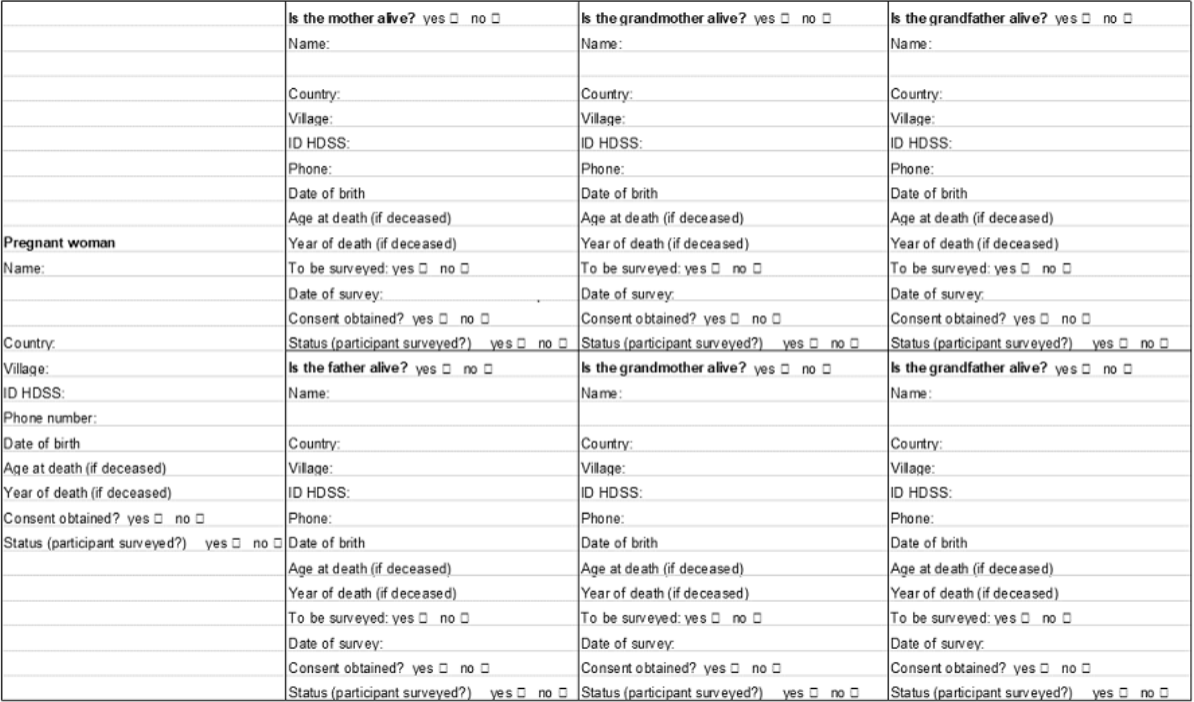
Family tree of the index women of the Taabo Multigenerational Cohort project. HDSS: Health and Demographic Surveillance System.

Once a pregnant woman is identified, she is visited in person by study staff and invited to participate in the study. Once written informed consent has been obtained, information about all the biological ancestors (father, mother, grandparents, and great-grandparents) of the indexed pregnancy or child (if recruited postpartum) is obtained and documented in a family tree form. This family tree form provides basic demographic information about the child’s relatives and gives details on which of them are still alive in the study area and therefore eligible for the study (Figure 4). The family tree document is reviewed and completed as interviewers receive additional information during the recruitment and interviewing of other family members. The family tree document is then used to recruit additional family members. These family members (fathers, grandparents, or great-grandparents of the index child) will then be visited in person and enrolled into the study conditional on their written informed consent.

### Study Staff

The field enumerators were recruited and trained for this study and work full-time to establish the cohort baseline. Their role is to establish and maintain contacts with village leaders, health centers, and study participants. In each study village, an information meeting was held with key informants at the beginning of the study. These meetings were organized in collaboration with the village authorities and representatives of the health services (hospital and health centers). After the initial meeting, study field enumerators kept in contact with health services in order to obtain information on newly registered pregnant women. Births were recorded from household visits and local health centers and hospitals to identify recent births and enroll new mothers and their babies.

Furthermore, in the course of their duties, study field staff may sometimes be confronted with complaints or refusals due to the length of the interview. In order to respect the participant’s wishes and reduce the risk of unfinished interviews, we negotiate an additional appointment with the participant whenever necessary. It can also happen that participants overlap because they belong to the family trees of different families at the same time. Similarly, interviewers may forget to carry out a household survey for a given participant. All these errors are detected and corrected thanks to weekly internal consistency checks in each data file and external consistency checks between the different data tables.

### Data Collection Instruments

#### Overview

The Taabo MGC uses 3 main questionnaires: an adult questionnaire, a late enrollment questionnaire, and a household questionnaire. The adult form contains 3 main blocks of questions: a core block of questions that concerns all participants without exception, a second block of aging-related questions that are asked only to persons aged 50 years or older, and a third block of maternal and child health–related questions that are only asked to women with current or recent pregnancies.

Table 1 gives an overview of data collection tools used for the Taabo MGC.

**Table 1.** Overview of data collection tools for the Taabo multigenerational birth cohort.

Questionnaire sections	Adults’ age (<50 y)	Pregnant women	Women	Adults’ age (≥50 y)	Adults’ age (≥60 y)	Adults who are disabled
Demographic characteristics	✓	✓	✓	✓	✓	✓
Mental health (depression, anxiety, stress, posttraumatic stress disorder): PHQ-9^a^, GAD-7^b^, and stress scale	✓	✓	✓	✓	✓	
Resilience	✓	✓	✓	✓	✓	
Personality assessment	✓	✓	✓	✓	✓	
Behavioral measurements (tobacco and alcohol; STEPS^c^)	✓	✓	✓	✓	✓	
General diet	✓	✓	✓	✓	✓	
Dietary salt	✓	✓	✓	✓	✓	
Raw food consumption	✓	✓	✓	✓	✓	
Handwashing practices	✓	✓	✓	✓	✓	
Physical activity	✓	✓	✓	✓	✓	
Cognition and memory test	✓	✓	✓	✓	✓	
Pattern recognition	✓	✓	✓	✓	✓	
Environmental exposure	✓	✓	✓	✓	✓	
Sibling and parent mortality	✓	✓	✓	✓	✓	
Health history (diabetes, hypertension, cholesterol, disability, and cardiovascular disease)	✓	✓	✓	✓	✓	
Quality of life	✓	✓	✓	✓	✓	
Physical examination	✓	✓	✓	✓	✓	
Instrumental activities of daily living scale				✓	✓	
Hasegawa dementia scale					✓	
Physical performance (balance, gait speed, and strength tests)				✓	✓	
Cervical cancer screening		✓	✓			
Obstetric history		✓	✓			
Breastfeeding initiation, exclusivity, and duration		✓	✓			
Antenatal care		✓	✓			
Short form						✓
Physical measurements (height, weight, waist and hip circumference, leg and trunk length, blood pressure, and heart rate)	✓	✓	✓	✓	✓	
Malaria rapid diagnostic test	✓	✓	✓	✓	✓	
Hemoglobin	✓	✓	✓	✓	✓	

a PHQ-9: Patient Health Questionnaire-9.

b GAD-7: General Anxiety Disorder-7.

c STEPS: World Health Organization’s STEPwise Approach to Surveillance.

#### All Women, Including Pregnant Women

The sections of the questionnaire completed by all women included questions on their prepregnancy obstetric history (cesarean section, miscarriage, stillbirth, live birth, and abortion experience; breastfeeding; place of delivery, child’s weight at birth; child’s civil registration; and current school attendance). Only the index woman was asked about their current pregnancy experience (their weight in kilograms, antenatal care attendance, use of supplements and antenatal services, and plans for delivery). If enrollment occurred after birth, a late enrollment questionnaire similar to the adult form was completed.

#### Adult Family Members

The core modules collected from all adults build heavily on the validated CoDuBu cohort questionnaire and health examination [11]: (1) study information and consent for participation (including pregnant women and covering their unborn children) and (2) sections on sociodemographic characteristics; behavioral measurements of tobacco use, alcohol consumption, diet, and physical activity; health history; anthropometric measurements such as blood pressure, height and weight, and waist circumference; questions from the World Health Organization’s STEPwise Approach to Surveillance to chronic disease risk factor surveillance [30]; health examination findings (blood pressure and pulse, gait speed, hemoglobin, and malaria infection assessment); self-rated general quality of life [31]; parents’ survival history and siblings of the adult; depression, anxiety, stress, and resilience score, based on the Patient Health Questionnaire-9 [32], the Resilience scale [33], Generalized Anxiety Disorder Screener [34], and the Depression Anxiety Stress Scale [35]; and cognition measured through the digit span [36] and progressive matrices tasks [37]. When the adult is not cognitively capable of completing the interview, a few basic questions (gender, date of birth, and cause of disability) are addressed to the person caring for the disabled participant. The question on cervical cancer screening was only asked to women.

#### Special Module for Older Adults (Aged 50 Years and over)

Individuals aged 60 years and above are asked to complete three additional aging-related modules: (1) Brody Instrumental Activities of Daily Living Scale [38], (2) the Revised Hasegawa’s Dementia Scale for cognitive function assessment [39], and (3) the Short Physical Performance Battery to assess physical fitness. The Short Physical Performance Battery covers 3 main areas: balance (standing in 3 positions), gait speed (walking speed at a normal pace), and strength (standing up and sitting in a chair) [40].

#### Household Questionnaire

The household survey questionnaire captures additional household background information, including household size; source of drinking water; lighting mode; toilet facilities; mosquito net use; main material of household roof, walls, and dwelling floor; wealth and assets; cooking mode; and agricultural and livestock property. We will only collect one household form for households hosting multiple study participants.

### Planned Fieldwork

During the first phase of this study (that we focus on in this protocol), the main goal is to enroll all index women and their families into the study. As part of this first phase—scheduled to end in June 2026—we will also conduct a short follow-up with women approximately 6 months after delivery to confirm pregnancy outcomes and verify basic childbirth characteristics (location, weight, and survival).

We aim to track cohort survival through the Taabo HDSS, which will collect basic sociodemographic information (survival, marriage, schooling, and employment) for all local residents at least annually in the foreseeable future. Additional future follow-up rounds could be used to collect additional behavioral or medical data; we also see this cohort as a platform to test specific interventions.

### Retention of Participants

To minimize the risk of participant attrition, we established a retention framework based not only on the collection of contact information but also on compensation to encourage continued engagement. Each participant is asked to provide their full name, phone number, the name of the household head, and the full name and phone number of the pregnant woman to whom the participant is linked, as well as the household ID and geographic coordinates. Furthermore, participants receive a summary report capturing the results of their physical examination, including blood pressure, height, and weight, as well as hemoglobin levels and *Plasmodium* infection for participants consenting to these tests. Results are explained in person and written on the summary report. If the test results suggest hypertension, anemia, or *Plasmodium* infections, participants are advised to go to their nearest health center for management. Moreover, at the end of the interview, each participant receives a gift of 2 pieces of soap worth around US$ 2.

Participation is voluntary, and participants may withdraw from the study at any time or may refuse to answer any questions without having repercussions on their participation. If the participant decides to withdraw from the study, they may request that their information already obtained be destroyed and electronic data be deleted and not be used for evaluation.

### Data Management

The interview data are collected by field enumerators using portable electronic devices (tablets) with incorporated data entry forms. Data collection is done using the Open Data Kit (ODK) package. Data storage is on a secure server at the Swiss Tropical and Public Health Institute (Swiss TPH) using the ODK Aggregate server and secured via SSL. This server hosts questionnaires and collected data. Once the data are uploaded on the server, they become accessible through the web interface of ODK Aggregate after the user logs in to the system.

### Statistical Analyses

The study will provide descriptive epidemiologic statistics on children and their families’ current health and health issues, including incidence and prevalence estimates, disease geospatial distributions, the intergenerational distribution, and determinants of common health risks and diseases of public health relevance to the Taabo department. Cox proportional hazards models will be used to analyze survival as the primary outcome of the study. Associations between outcomes and explanatory variables will be tested using multivariable statistical analysis, with statistical significance set at *P*<.05. Logistic regression models will be used to analyze binary outcomes, and ordinary least squares linear regression models will be used to analyze quantitative variables of interest. All statistical analyses will be performed using Stata software (version 19; StataCorp College Station). Missing data will be handled using multiple imputation to replace missing values.

In order to provide a sense of how comparable this cohort is to the general population of Côte d’Ivoire, we compare average characteristics of pregnant women as well as enrolled households to those reported in the latest nationally representative household survey, which is the Demographic and Health Survey collected in 2021.

## Results

As of December 2024, a total of 3239 women and 6501 family members were enrolled in the Taabo MGC. The enrollment of families will continue until December 2025, with the aim of covering a sample of at least 15,000 participants.

Table 2 compares the data collected to date to the 2021 Côte d’Ivoire DHS. The pregnant women are overall quite similar to the national average, with a higher share of pregnant women aged less than 20 years and a slightly lower share in the age range between 25 and 29 years. In terms of educational attainment, women in the cohort are more likely to have attained primary education and less likely to not have attended school or attended college. All of the biomarkers collected—height, weight, and hemoglobin levels—are remarkably similar to the national averages.

At the household level, average asset ownership is also fairly similar, with almost identical rates of water access, TV ownership, and radio ownership. Compared to the national average, there is substantially lower access to improved sanitation (as well as much higher rates of open defecation) and increased ownership of bicycles and motorbikes.

**Table 2.** Comparison of the Taabo Multigenerational Cohort participants and national averages.

Pregnant women			Taabo MGC^a^^,b^		Côte d'Ivoire 2021^c^	
**Age (y), n (%)**						
15‐19			768 (21.3)		92 (13.5)	
20‐24			971 (26.9)		191 (28.0)	
25‐29			707 (19.6)		171 (25.0)	
30‐34			574 (15.9)		124 (18.1)	
35‐39			426 (11.8)		75 (11.0)	
40‐44			146 (4.0)		22 (3.3)	
>45			16 (0.4)		8 (1.2)	
**Education, n (%)**						
No education			1659 (46.0)		380 (55.6)	
Primary education			1059 (29.4)		147 (21.5)	
Secondary education			857 (23.8)		131 (19.2)	
Higher education			33 (0.9)		24 (3.6)	
**Marriage status, n (%)**						
Never married			1133 (31.4)		103 (15.1)	
Married			1340 (37.1)		575 (84.2)	
Divorced			28 (0.8)		5 (0.7)	
**Patient characteristics**						
Height (cm), mean (SD)			159.4 (6.1)		159.6 (5.8)	
Weight (kg), mean (SD)			62.1 (11.4)		61.9 (11.9)	
BMI (kg/m^2^), mean (SD)			24.4 (4.1)		24.3 (4.2)	
Hemoglobin, mean (SD)			100.7 (14.0)		103.2 (14.8)	
Anemic, n (%)			2615 (72.5)		417 (65.9)	
**Household characteristics, n (%)**						
Has piped water			1763 (36.6)		5363 (36.3)	
Has a flush toilet			872 (18.1)		5311 (36.0)	
Has no toilet at all			2469 (51.3)		2689 (18.2)	
Has a radio			2157 (44.8)		6900 (46.7)	
Has a television			2443 (50.8)		8204 (55.6)	
Has a fridge			756 (15.7)		2953 (20.0)	
Has a bicycle			2717 (56.5)		3619 (24.5)	
Has a motorbike			2051 (42.6)		4538 (30.7)	
Has a car			104 (2.2)		722 (4.9)	

a On the basis of data collected up to June 18, 2025.

b MGC: Multigenerational Cohort.

c On the basis of Côte d’Ivoire Demographic and Health Survey 2021.

## Discussion

### Anticipated Findings

This paper describes the design of a large, regionally representative multigenerational cohort within the Taabo HDSS that will help identify appropriate interventions and guide health policies at the country and regional levels. To our knowledge, this is the first birth cohort study that is being carried out within an active HDSS area to assess the links between exposure factors and the occurrence of health events starting in early childhood. The data from this study can further strengthen local research infrastructure and open new opportunities for global comparative epidemiology. Ultimately, we hope to enroll at least 3000 pregnant women, including their children, the children’s fathers, and their grandparents and great-grandparents, to reach a sample of at least 15,000 participants. Births of interest to the Taabo MGC are those of children born during the 24 months following January 1, 2024. To date, 6655 eligible participants have been registered from the enrollment of 2502 women (n=2146, 85.8% during pregnancy and n=356, 14.2% post partum), equivalent to an average of around 3 participants per family. This is well short of the theoretically possible 14 family members illustrated in Figure 3 (2 parents, 4 grandparents, and 8 great-grandparents). There are several reasons contributing to the unexpectedly small family sizes recruited so far. First, in some cases, pregnancies are not recognized by fathers, making it impossible to recruit the paternal side of the family. Second, the proportion of adults living beyond the age of 60 years has been low historically, limiting the number of grandparents and great-grandparents alive today [2]. Third, and maybe most importantly, the Taabo area is home to a large population not originating from this region, including large Burkinabe and Malian communities that settled there in the late 1970s when the Taabo hydroelectric dam was built [41]. The fishing opportunities in the lake created by the dam, as well as the suitability of the local soil and climate for cocoa farming, have also resulted in substantial in-migration from other regions of Côte d’Ivoire as well as neighboring countries, frequently separating families with young children from their parents and grandparents.

The relatively small size of local families is a first finding of this cohort and a societal aspect to be investigated in future studies.

Given that the entire population of the study area is under surveillance, we are confident that we can trace the cohort well over time and minimize the risk of loss to follow-up. We are confident that the cohort will provide key data on local population health, such as the prevalence of risk factors and morbidity in the short run. In the longer run, we hope that the Taabo MGC can provide a broad platform for future local observational and interventional studies, promote international collaborations and consortia, and contribute to the much-needed evidence base on lifetime disease risk in SSA. We also hope to use this cohort as a resource to create policy-relevant findings and hope to be in regular exchange with local policy makers to share insights from this cohort. While the cohort is only representative of a very specific region of the country, the data presented here suggest that the population under surveillance is rather similar to the national average in many respects.

### Conclusions

We trust that the new Taabo MGC cohort will provide ample opportunities to study health behaviors and outcomes across generations in a rural African setting. Recruitment into the cohort should be completed by the end of 2025—we hope to follow participating families for many years through the established and productive HDSS system.

## Supplementary material

10.2196/70771 Multimedia Appendix 1 Medical information sheet.

## Abbreviations

CD: communicable disease
CoDuBu: Côte d’Ivoire Dual Disease Burden Cohort
HDSS: Health and Demographic Surveillance System
MGC: multigenerational cohort
NCD: noncommunicable disease
ODK: Open Data Kit
SSA: sub-Saharan Africa
