# Supplementary figures and images for: Taabo Multigenerational Birth Cohort in Côte d'Ivoire: Protocol for Establishing a Longitudinal Multigenerational Birth Cohort to Guide Health Policy
Source: JMIR Res Protoc. 2025 Oct 7;14:e70771. doi: 10.2196/70771 (PMC12505403; doi:10.2196/70771)

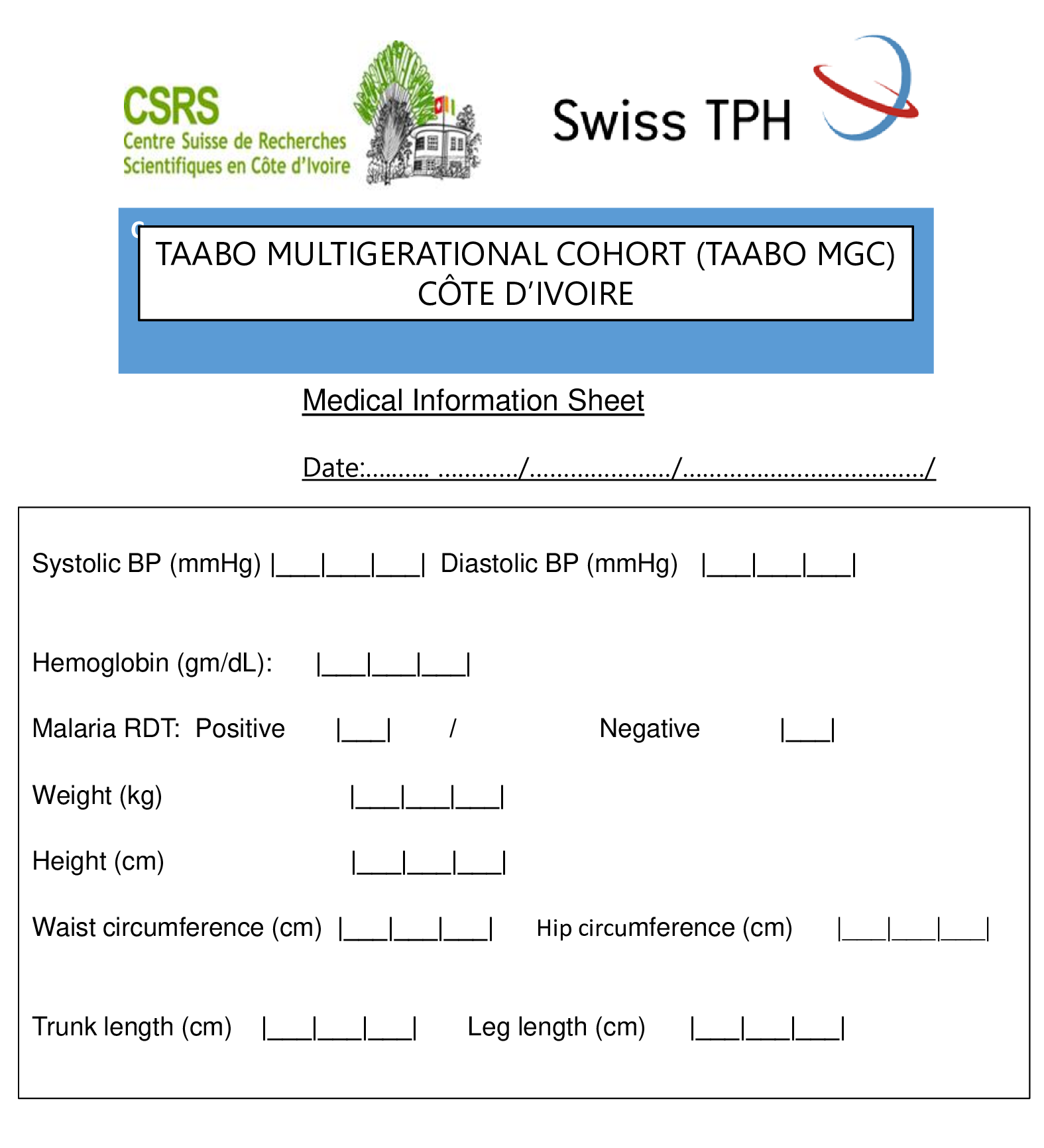

Supplement: Multimedia Appendix 1 [file resprot-v14-e70771-s001.png]
